# Digital empowerment of women in the South African public sector

**DOI:** 10.3389/fsoc.2025.1604857

**Published:** 2025-10-21

**Authors:** Elvin Shava, Tigere Paidamoyo Muringa

**Affiliations:** College of Law and Management, University of Kwazulu-Natal, Durban, South Africa

**Keywords:** digital empowerment, women in public sector, digital disparities, South Africa, digital governance

## Abstract

**Introduction:**

The digital empowerment of women in the South African public sector presents a strategic opportunity to enhance women’s technical competencies and boost their confidence in digital environments. Despite global progress, the inclusion of women in digital technologies and information communication technologies (ICTs) in South Africa remains low, reflecting persistent gendered marginalisation in the digital economy.

**Methods:**

This study employed a systematic literature review methodology to investigate the challenges and opportunities surrounding women’s digital empowerment in the public sector of South Africa. Scholarly databases and search engines, including Google Scholar, Web of Science, and ScienceDirect, were used to retrieve and analyse peer-reviewed publications and relevant grey literature.

**Results:**

The findings highlight significant digital disparities that hinder the empowerment of women in the public sector. Key barriers include limited access to technical skills training, insufficient funding, a lack of targeted skills development workshops, organisational resistance to change, and broader issues of data marginalisation. These factors collectively undermine women’s participation in the digital transformation of public service.

**Conclusion:**

The study emphasises the urgent need for government-led initiatives to address the digital skills gap among women in the public sector. Strategic coproduction between government and stakeholders is essential to ensure inclusive and sustainable digital empowerment programmes. This research adds to the body of knowledge on digital democracy, innovation, and the empowerment of marginalised groups within public sector transformation efforts.

## 1 Introduction

Digital empowerment of women in South Africa is crucial for reducing gender inequality. It also supports economic and social progress (Aslam et al., 2024). The Constitution of the Republic of South Africa (1996) and key laws, such as the Promotion of Equality and Prevention of Unfair Discrimination Act (2000), the Employment Equity Act, and the Broad-Based Black Economic Empowerment Act (2003), call for women’s active participation in digital and ICT spaces. This includes roles within the public sector. These laws aim to ensure equity, gender inclusion, and the growth of digital skills. Government and private sector support is vital (Ojo, 2022). Their involvement helps shape and implement policies that close the digital divide. These efforts also boost women’s engagement in the digital economy (Hendricks and Olawale, 2022).

Despite the implementation of numerous policies, gender gaps remain a serious concern in access to digital resources and information and communication technology (ICT) literacy within South Africa’s public service system (Maleka, 2011; Van Rheede, 2023). While some research highlights digital transformation in occupational settings, such as Van Rheede’s (2023) study of ICT professionals, others, like the studies by Chisango and Marongwe (2021), focus on the issue of digital exclusion in Quintile 1 schools, illustrating the context-specific challenges of accessibility. Both examples show how the boundaries between service delivery and formal workplace spaces tend to overlap, but also highlight the need to clarify the research scope. This study focuses on women as citizens, specifically those in marginalized conditions, who seek to access public digital services such as healthcare, education, social grants, and administrative systems. Many of these women face obstacles related to limited digital literacy, financial hardships, and prevailing socio-economic norms that restrict their access to and benefit from digital advancements in public service delivery (Chisango and Marongwe, 2021; Aruleba and Jere, 2022). The partial implementation of the national digital inclusion policy, coupled with the ongoing impact of social norms, hampers women’s full participation in South Africa’s digital democracy and transformation (Mare, 2021; Shava, 2021). Efforts to address key priorities for national development, as outlined in the National Planning Commission (2012), such as women’s inclusion in the digital economy and access to government services, have not yet solved these systemic issues.

South Africa is undergoing a significant digital development shift. Women, however, remain underrepresented in this process. Although mobile phones are widespread, women lack the digital literacy and institutional support needed to fully benefit from innovations in the public sector. This research is necessary because existing studies mainly focus on women in the private sector or on general ICT policies that do not sufficiently address women’s experiences in the public sector. As the country advances its strategies like the Department of Communications and Digital Technologies (2020), and tackles the Fourth Industrial Revolution, it becomes increasingly important to understand how women in public organizations are being enabled, or left behind, by digital initiatives. The After Access 2022–2023 Report offers encouraging trends: the gender gap in internet usage shrank from 8% in 2012 to nearly zero in 2022. However, access alone does not guarantee empowerment. Reports from the International Telecommunication Union, UNESCO, and the Gender Equality Index reveal ongoing disparities in digital skills and access to formal digital jobs. National strategies, such as the Department of Higher Education and Training (DHET) (2022) report on Economic Reconstruction and the Presidential Commission on the Fourth Industrial Revolution, also emphasize the need for inclusive participation in the digital economy. Women—particularly in public sector roles—continue to be marginalized in both implementation and benefit. This disconnect is what the study aims to explore.

Studies indicate that, amidst increasing attention to digital empowerment, most works have focused on women’s issues in the private sector, paying scant attention to the public sector. For example, Nesaratnam and Singh (2018) examined the gender digital divide in the IT sector and discovered significant barriers for women. In another related study, Hendricks and Olawale (2022) investigated the use of ICTs in education for women’s empowerment but did not elaborate on challenges faced within the public sector. On the other hand, studies on women’s digital empowerment within the South African public sector have been mostly descriptive, providing little insight into what hinders women from participating in digital transformation programs. Consequently, very little is known about how effective policies can be designed and implemented to foster women’s digital inclusion in public institutions. Shopola et al. (2023) therefore aim to fill this gap by exploring the perceived barriers to and facilitators of women’s digital empowerment in South Africa’s public sector, and provide informed recommendations based on evidence for the development of inclusive policies.

This review employs the PRISMA method to examine factors promoting and hindering digital empowerment among women in South Africa’s public sector. It draws on Empowerment Theory (Zimmerman, 2000) and the Diffusion of Innovations Theory (Rogers et al., 2014) to identify the main drivers of the digital gender gap. The study explores how government interventions, stakeholder coordination, and policy implementation can enhance women’s participation in digital training programs.

The purpose of the study is to critically analyze the barriers and facilitators that influence women’s digital empowerment in the South African public sector. The study specifically examines the role of institutional practices, policy frameworks, and stakeholder collaboration in women’s participation in digital transformation initiatives. It focuses solely on women who access digital public services daily. Through a systematic literature review, the research explores structural inequalities, implementation challenges, and possible inclusive digital policy opportunities. By concentrating on digital service access environments, the study offers a targeted gendered digital inclusion analysis, separate from broader societal trends, with implications for policy reform, capacity building, and gender-responsive governance. Two key questions are addressed:

- What are the key barriers to digital empowerment for women in the South African public sector, and how do factors such as limited funding and resistance to change contribute to these challenges?
- How can government interventions and stakeholder collaboration improve women’s inclusion in digital skills development programs within the South African public sector?

Following this introduction, the paper reviews the existing literature on digital gender disparities, barriers to women’s participation in ICT, and relevant policy frameworks. The methodology section describes the systematic review process for collecting and analyzing data from relevant studies. The findings section highlights key themes and factors that influence digital empowerment. The discussion interprets these findings within the frameworks of empowerment and diffusion of innovations theories. Finally, the conclusion offers recommendations to policymakers and practitioners on promoting women’s inclusion in digital initiatives.

## 2 Overview of the digital landscape in the south African public sector

Digital transformation has been taken up as a central pillar of service delivery reform in South Africa’s public sector. However, profound digital inequalities persist, particularly when viewed through a gendered and socio-economic lens. The government’s strategic plan, South Africa’s Roadmap for the Digital Transformation of Government, outlines a high-level commitment to digitising services through initiatives such as digital identity systems, interoperable data platforms, and digital payment systems (Republic of South Africa, 2023). While such reforms represent significant advances, a closer examination of the evidence through the lens of Helsper’s (2021) Corresponding Fields Model—adopting access, digital skills, and use, and outcomes- illustrates a multidimensional digital divide. Women, particularly those in rural areas, continue to be disproportionately excluded from the benefits of digital governance, a problem that requires intersectional, data-driven, and gender-sensitive solutions.

The first level of the digital divide—connection and infrastructural access—has made significant progress, but exclusion persists. In January 2025, 50.8 million internet users in South Africa, representing 78.9% internet penetration, were recorded compared to 74.7% in 2024 (HelloYes Marketing, 2025). Furthermore, 124 million mobile connections served 193% of the population, indicating widespread mobile penetration and multi-SIM usage (HelloYes Marketing, 2025). Nevertheless, approximately 13.6 million South Africans remain offline, most of them in remote rural communities where broadband infrastructure is inadequate. Although 91% of active mobile phones were smartphones (DHL South Africa, 2024), ownership gaps are evident, with women in impoverished communities often possessing non-smart mobile phones or relying on borrowing. These infrastructural deficiencies limit their access to essential digital services such as online education, health portals, or government support. The evidence suggests that digital access is less about availability and more about affordability, gender equality, and device quality—key indicators of the first level of the digital divide (Helsper, 2021).

The third level of the digital divide, unequal outcomes, shows how structural disadvantages lead to limited benefits for women in the digital age. As South Africa’s digital economy is projected to exceed R500 billion in GDP by 2026 (Digital Virgo, 2023), women’s participation remains negligible. Women hold only 21% of executive ICT roles and comprise just 18% of ICT graduates in the country (Matotoka and Odeku, 2021a, 2021b). Furthermore, women-owned digital businesses make up less than 10% of all new ventures in the sector (Digital Virgo, 2023). Financial exclusion worsens the problem: only 14% of women entrepreneurs use digital finance channels, compared to 32% of their male counterparts (Digital Virgo, 2023). As Helsper (2021) argues, even when access and skills gaps are reduced, unequal social positioning still limits women’s ability to gain sustainable economic and civic benefits from their digital engagement. Without structural measures to distribute digital opportunities more evenly, these gaps will persist.

Institutional impediments make the landscape even less clear. Despite progressive strategies such as the National Integrated ICT Policy White Paper and the Digital Economy Masterplan, provincial and municipal levels of implementation are partial (Venter et al., 2023). A mere 84 of the 257 municipal districts of South Africa have within them adequate digital inclusion infrastructure, and most of those are deficient in gender-sensitive programming (Venter et al., 2023). More troubling, gender-disaggregated digital indicators are scarce, thus constraining effective monitoring and assessment of inclusion. The *ad hoc* community centers and workshops in place presently through the vehicle of NGOs--as applicable--cannot make up for coordinated, state-led investment in digital equity. For digital transformation to reach its potential, work is needed that is coordinated throughout the ministries of government, that is predicated on the use of real-time data, and predicated on gender-responsive delivery and design.

Overall, the public sector’s digital transformation in South Africa, promising as the direction is, is nevertheless stymied by entrenched digital exclusions. Utilization of Helsper’s (2021) framework illustrates that exclusion occurs not only at the connectivity level but also at the capability and outcome levels. The digital world is also highly stratified along the dimensions of gender, geography, and socio-economic status. In bridging the gap, South Africa must make investments in balanced infrastructure addition, digital literacy programming among the marginalized, and outcome-driven reform anchored in outcome-directed targeting of women’s inclusion in the digital economy. Unless such conscious effort is made, digital transformation is sure to deepen, rather than eliminate, the very target it aims to eradicate.

## 3 Methodology

### 3.1 Study design

This study adopted a systematic review approach informed by the PRISMA framework for conducting a systematic review. This systematic review synthesises existing literature on women’s digital empowerment in South Africa’s public sector. It identifies barriers, facilitators, and gaps in women’s digital empowerment, as noted in previous research.

### 3.2 Study strategy

This systematic review requires a very careful development of a search strategy to make sure that all the studies fitting its criteria for the digital empowerment of women in South Africa’s public sector are included (Muringa and Shava, 2025). The use of various academic databases, including Web of Science, Scopus, Google Scholar, and EBSCOhost, was employed to gather the broadest possible search results on research related to gender studies, digital inclusion, and public sector development. This was a targeted search to identify peer-reviewed articles published between 2010 and 2025.

A list of well-researched keywords guided the search. They included, amongst others, “digital empowerment of women,” “gender digital divide,” “ICT access,” “public sector,” and “South Africa.” Boolean operators such as AND and OR combine these keywords, allowing the search to be both narrow and encompassing enough to bring up many studies on the topic. The search terms were adapted where needed to suit the requirements of each database. The search strategy also included government reports from the South African government’s official websites and academic databases, as well as international development agencies that deal with gender and ICT issues.

#### 3.2.1 Inclusion and exclusion criteria

Inclusion and exclusion criteria were used to ensure that only relevant studies were included in the review. The study included peer-reviewed articles written in English from 2010 to 2025 that focused on the digital empowerment of women in South Africa’s public sector. Besides government reports, all non-peer-reviewed works, opinion pieces, editorials, and studies that only targeted the private sector or did not address gender and digital empowerment were excluded.

### 3.3 Data collection procedure

**Figure 1:**
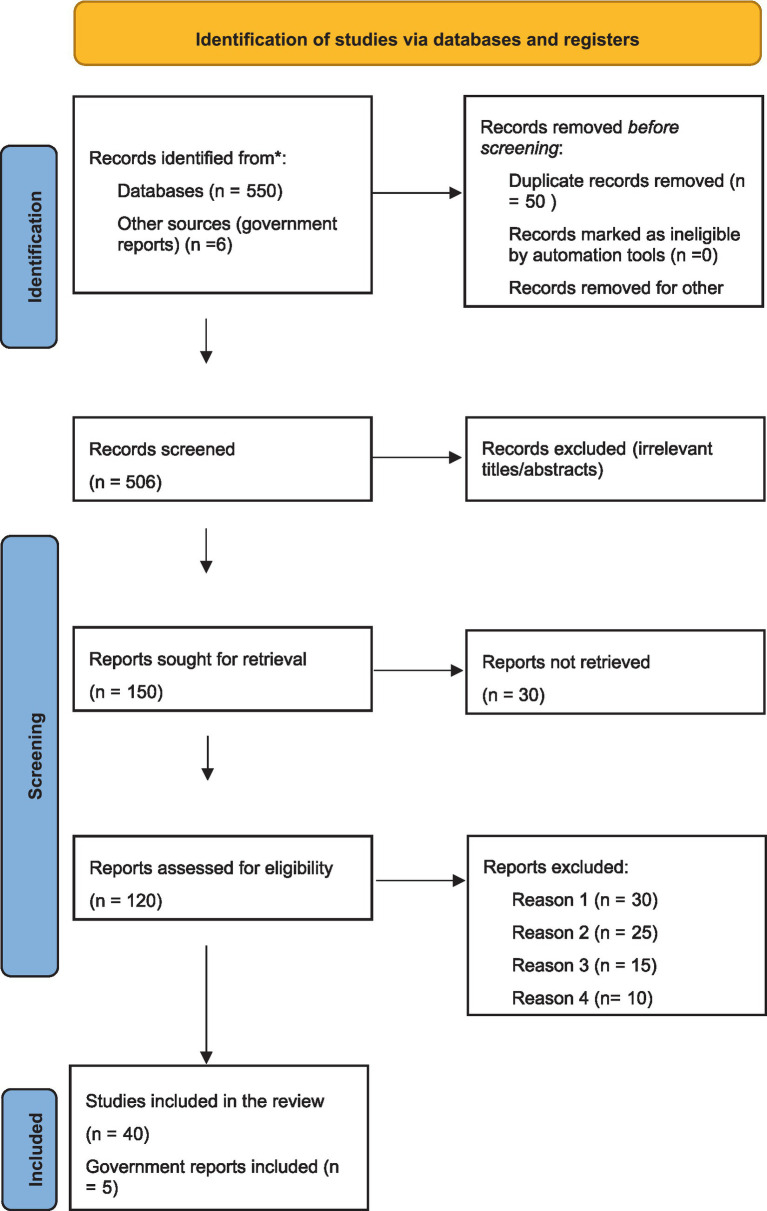
PRISMA diagram.

From the selected databases and other sources, 550 records were identified, in addition to 6 from government reports, totaling 556. Records retrieved at each stage of this process were summarized in Figure 1. Fifty duplicate records were removed, leaving 506 unique records to be screened. After eliminating irrelevant articles by reviewing titles and abstracts, 356 records were excluded, which narrowed the focus to women’s digital empowerment within the South African public sector. The remaining relevant articles numbered 150, plus 6 government reports, making a total of 156 records for further full-text analysis.

During the full-text review, 36 records were excluded because they focused on unrelated sectors like the private sector or education. Some were purely theoretical and lacked empirical evidence. Others did not provide specific analysis on gender differences in digital empowerment. In total, 120 records, including government reports, were retained for further assessment of eligibility.

After a thorough assessment, 40 peer-reviewed studies and five government reports were ultimately included in the systematic review. A spreadsheet was used to code data from the selected articles, which included information such as the author’s name, article title, journal name, publication year, geographic context, sample size, gender composition, and main findings on digital empowerment. Various reasons led to their exclusion, including narrow or focused case studies, reviews, and studies that did not explicitly address digital empowerment or were not focused on the public sector. The steps of the systematic review are shown in Figure 1. After examining the articles against the inclusion and exclusion criteria, 40 articles were selected for inclusion in this study.

#### 3.3.1 Coding

Thematic synthesis coding started with a thorough and systematic review of the literature, where eligible relevant works, including peer-reviewed articles and government reports, were screened for relevance. Following Strauss and Corbin’s coding method from grounded theory, researchers performed open coding to develop initial codes like “limited internet access,” “gender bias,” and “policy implementation gaps.” These codes were directly extracted from quotes in the literature and served as foundational data units. The iterative and comparative process in this phase allowed researchers to recognize recurring patterns of facilitators and barriers to digital empowerment of women in South Africa’s public sector.

Axial coding then grouped similar codes into broader conceptual categories like “infrastructural barriers,” “patriarchal exclusion,” and “weak legislative enforcement.” Selective coding then refined these categories, merging them into higher-level themes such as “Policy and Legislative Barriers,” “Institutional and Cultural Exclusion,” and “Stakeholder Intervention Gaps.” These emerging themes directly addressed the research questions, capturing the structural, political, and socio-economic factors impacting digital empowerment. The visual model below illustrates this layered, step-by-step analytical process that ensured empirical accuracy and clear themes.

### 3.4 Findings

The presentation and discussion of findings are organized around the two primary research questions that guided this study. The first question examined the primary barriers, such as funding, resistance to change, and socio-cultural factors, that hinder women’s access to and use of digital technologies in efforts toward digital empowerment in the South African public sector. It also addressed broader systemic challenges that contribute to the digital gender divide. The second question examined how government and stakeholder efforts, through policy, infrastructure, and training, facilitate women’s inclusion in digital skills programs and the digital economy. These discussions aim to provide insight into how strategic initiatives can help bridge the gender digital divide and promote empowerment in the public sector.

#### 3.4.1 Summary of included studies

This review included 40 peer-reviewed articles and 5 government reports that offered insights into women’s digital empowerment in the South African public sector. The studies covered various regions of South Africa and used both qualitative and mixed-method approaches, with sample sizes ranging from 50 to 300 participants. Most studies used interviews, case studies, and surveys to examine the barriers and enablers of digital empowerment. The focus was mainly on urban areas, but some studies also looked at rural populations, highlighting socio-economic and infrastructural gaps.

#### 3.4.2 Policy implementation and legislative barriers

Despite the existence of progressive legislative measures in South Africa aimed at advancing gender equality and digital inclusion, implementation remains inconsistent and largely ineffective, especially in rural areas (Antonites, 2021; Omweri, 2024). The issue is not the lack of laws, but the persistent gap between policy creation and implementation (Shava, 2021). For example, while the National Development Plan (NDP) explicitly details strategies for promoting digital skills among marginalized groups, its implementation in rural regions remains inadequate (Mwansa et al., 2025), further deepening existing digital divides (Nesaratnam and Singh, 2018). Multiple studies confirm that legal mandates intended to improve women’s access to ICT are often poorly enforced (Lewis, 2013), particularly at the local government level, where administrative capacity and oversight are weakest (Nesaratnam and Singh, 2018).

Key legislative frameworks, such as the Constitution of the Republic of South Africa (1996), the Promotion of Equality and Prevention of Unfair Discrimination Act (2000), and the Electronic Communications Act (2005), establish commendable principles that support equal access to digital opportunities. However, these principles have not resulted in practical reforms or institutional change. Women continue to face significant barriers to accessing ICTs and developing skills (Matlala, 2025), particularly within public institutions, where outdated recruitment practices and a lack of accountability hinder inclusivity (Domínguez-Munllonch, 2023). The Employment Equity Act (1998) mandates affirmative action (Montesh, 2010). However, vague interpretations and weak enforcement have left women underrepresented in digital leadership and excluded from strategic decisions (Hendricks and Olawale, 2022).

Furthermore, the Broad-Based Black Economic Empowerment Act (2003), initially designed to address historical socio-economic inequalities, has primarily focused on racial equity at the expense of gender-specific redress (Espi et al., 2019). As a result, women, especially those in rural and peri-urban areas, have been underrepresented in digital skills development programs, exacerbating their marginalization in the digital economy (Chisango and Marongwe, 2021). Likewise, the National Integrated ICT Policy White Paper (2016), which seeks to close digital access and literacy gaps, has not produced meaningful benefits for women in underserved areas (Munyoka, 2022). Its slow rollout and absence of gender-focused interventions have limited its ability to change women’s digital participation (Domínguez-Munllonch, 2023).

Furthermore, the Broad-Based Black Economic Empowerment Act (2003), originally designed to address historical socio-economic inequalities (Munkuli, 2024), has primarily focused on racial equity at the expense of gender-specific redress. As a result, women, especially those in rural and peri-urban areas, have been underrepresented in digital skills development programs (Katunga et al., 2023), exacerbating their marginalization in the digital economy (Chisango and Marongwe, 2021). Similarly, the National Integrated ICT Policy White Paper (2016), which aims to close digital access and literacy gaps (Nyahodza and Higgs, 2017), has not produced meaningful results for women in underserved areas. Its slow implementation and lack of gender-focused interventions have limited its ability to significantly improve women’s digital participation (Domínguez-Munllonch, 2023).

#### 3.4.3 Political barriers

Political instability also serves as a key structural barrier to women’s digital empowerment in South Africa’s public sector (Alao et al. 2022). Frequent leadership changes often cause sudden shifts in policy priorities and reallocate budgets, disrupting long-term plans for digital inclusion (Manda and Backhouse, 2018). Mosala (2022) notes, political will diminishes as the national focus shifts to urgent crises like economic downturns or infrastructure issues. This instability weakens the continuity and effectiveness of digital empowerment initiatives, especially those designed with a gender-sensitive approach (Motloung, 2021). In many cases, budget allocations are withheld or redirected, leading to delays in implementation and reduced reach (Independent Communications Authority of South Africa (ICASA), 2025), particularly in rural areas where women face multiple socio-economic challenges (Motloung, 2021; Chisango and Marongwe, 2021).

Furthermore, the politicization of ICT programs often tends to overlook gender equality objectives in favor of broader political agendas that ignore the specific interests of marginalized women (Antonites, 2021). Research in similar contexts, including Nigeria and Mexico, reveals a similar pattern of gender-inclusive digital policies being sidelined during periods of political change and inconsistent governance (Okonjo-Iweala, 2021; Martinez and Ortega, 2021). Ineffective, long-term policy reports based on cross-sectoral consensus also restrict implementation. Adedokun and Zulu (2022) argue that successful digital empowerment requires long-term commitment. This includes not only government support but also inclusive governance to protect gender programs from political shifts. This emphasizes the need to strengthen institutional checks and multistakeholder accountability mechanisms that prevent women’s digital inclusion from being vulnerable to political risks (Independent Communications Authority of South Africa (ICASA), 2025).

#### 3.4.4 Institutional barriers

The institutional barriers in the public sector manifest as gender prejudices and discrimination within ICT departments, where women hold fewer leadership roles (Khwela et al., 2020). Research indicates that most women in the public sector frequently encounter institutional resistance when attempting to enter or advance in ICT-related positions (Nesaratnam and Singh, 2018). Many public organizations are led by a patriarchal culture that alienates women and hampers their training and development. Nkosi (2023) indicates that even in South Africa’s public sector, women are frequently excluded from decision-making processes involved in starting digital transformation, which prevents them from participating meaningfully in ICT development.

Patriarchal norms in South African society hinder women’s digital empowerment, as cultural expectations often restrict their ability to pursue ICT careers or participate in digital skills training (Nkosi, 2023; Shopola et al., 2023). Studies show that women, especially in rural areas, are frequently expected to prioritize family responsibilities over personal development (Shava, 2021), which limits their participation in digital learning programs (Nesaratnam and Singh, 2018). The digital gender divide is further exacerbated by societal attitudes that discourage women from entering male-dominated fields, such as ICT and digital technologies (Pokpas, 2019; Siwale and Mwalemba, 2023).

#### 3.4.5 Socio-economic environment of the public sector

The socio-economic environment in the South African public sector worsens the digital divide (Shiferaw, 2024), with women from lower-income backgrounds having limited access to the internet, digital devices, and ICT training (Niyitunga, 2024). The reviewed studies revealed that women in rural areas are especially disadvantaged. They are more likely to face poverty, unemployment, and limited educational opportunities, all of which contribute to their exclusion from digital empowerment initiatives (Frans and Pather, 2021). Additionally, the lack of affordable digital infrastructure, such as high-speed internet and data, further limits women’s ability to participate in the digital economy (Gillwald, 2024; Nkosi, 2023).

Despite challenges, several studies highlight potential opportunities for digital empowerment in the South African public sector. For example, the rise of digital platforms and mobile technologies can bridge the digital divide by giving women access to education, healthcare, and employment opportunities (Coachability Foundation, 2025; Statistics South Africa, 2022). Additionally, community-driven ICT centers have successfully provided women with basic digital literacy skills, although these initiatives often face financial constraints (Motloung, 2021; Frans and Pather, 2021). Studies also suggest that with proper funding and support, these opportunities could significantly increase women’s participation in the digital economy (Gillwald, 2024; Nkosi, 2023).

#### 3.4.6 Improving women’s inclusion in digital skills development programs

Enhancing women’s inclusion in digital skills development programs within the South African public sector requires coordinated efforts from the government and various stakeholders (Aslam et al., 2024), including private sector partners, non-governmental organizations (NGOs), and community-based organizations. Stakeholder partnerships are essential for improving digital skills development among women by addressing capacity gaps that government efforts alone cannot fill (Omweri, 2024). Collaboration among government agencies, private sector companies, NGOs, and international organizations has provided funding, infrastructure, and technical support for women’s digital inclusion (Adedokun and Zulu, 2022; Shopola et al., 2023). For instance, telecommunication companies have offered affordable internet access in impoverished communities, enabling better participation in online learning programs (Frans and Pather, 2021). NGOs play a key role in providing digital literacy training at the community level, especially in rural areas where government services are limited (Motloung, 2021). Beyond digital literacy, these organizations typically work to overcome socio-cultural barriers to participation through empowerment and mentorship programs grounded in women’s lived experiences (Hendricks and Olawale, 2022; Nkosi, 2023; Ojo, 2022).

However, coordination and sustainability remain ongoing challenges. Fragmented implementation, overlapping mandates, and poor alignment of goals among stakeholders often lead to inefficiencies and diminished impact (Chisango and Marongwe, 2021). Additionally, CSR initiatives by the private sector are usually short-term and have limited targets, lacking the capacity to address deep-rooted structural issues such as entrenched gender norms or unequal access to employment opportunities (Domínguez-Munllonch, 2023; Nesaratnam and Singh, 2018). Still, emerging evidence shows the potential of well-designed public-private partnerships that leverage private sector technical expertise and NGOs’ grassroots networks. These partnerships not only expand program coverage but also promote continuity and impact by providing sustained support and opportunities for career advancement for women (Mashologu, 2023; Frans and Pather, 2021). Such models can be scaled through joint planning and shared responsibility to establish inclusive digitalization across sectors.

## 4 Discussion

The evidence in this research indicates that South Africa’s digital gender gap in the public sector is not solely caused by technology, but is also rooted in institutional, policy, and socio-cultural structures that reflect regional trends across sub-Saharan Africa. The persistent gap between revolutionary laws and actual progress for women is a common issue in the region, where countries sign gender-inclusive agreements but fail to implement them due to political instability, inconsistent funding, and bureaucratic delays (Adeola, 2020). For example, while South Africa has faced challenges in implementing the National Integrated ICT Policy White Paper (2016), Kenya has encountered similar issues in executing its Vision 2030 agenda for digital inclusion, particularly for women in peri-urban areas (Ndungo and Ngugi, 2025). Women are often secondary beneficiaries of these policies, even when they are explicitly named as target groups.

Institutionally, women’s exclusion from ICT-related professional and leadership paths reflects deep structural disparities in the private and public sectors across in South Africa and beyond. As Irene et al. (2025) argue, technological progress has not always translated into empowerment; instead, without protective measures, digital advancements may reinforce existing hierarchies and even reverse previous gains made by women entrepreneurs (Kamberidou, 2020). The South African example mirrors these trends, where ICT centers and digital hubs are established with good intentions but fail to create systemic change due to the lack of professional ecosystems, mentorship pipelines, and employment integration mechanisms (Mhlanga, 2024). Furthermore, South Africa’s cross-sectoral inconsistencies are like findings in Ghana and Nigeria, where fragmentation among ministries, donors, and community-based organizations has hindered digital inclusion efforts (Kampini et al., 2023; Udenigwe and Yaya, 2022). In all these cases, the failure to embed ICT within broader gender and economic planning structures has limited women’s advancement from basic digital literacy to more complex digital work or entrepreneurship.

This article also emphasizes that digital empowerment is deeply connected to socio-cultural and economic circumstances. South African women’s digital access is limited not only by infrastructure issues but also by domestic responsibilities and social norms that dictate their time availability (Khumalo and Saurombe, 2022), concerns that Ndungo and Ngugi (2025) found particularly relevant for low-income and older women balancing informal childcare work in Kenya. Similarly, patriarchal expectations in South Africa’s rural areas have counterparts in Ethiopia and Uganda, where women’s digital adoption still depends on male approval or family consent (Niroo and Crompton, 2022). This suggests that digital policy, unless grounded in culturally sensitive and community-driven approaches, risks becoming tokenistic or exclusionary (Bui, 2025). The literature also confirms that even when women gain access to digital platforms, such as maternal health programs in Uganda and Tanzania, their participation remains uneven and is influenced by factors including education, mobility, and domestic power dynamics (Ogundaini et al., 2024; Abdul et al., 2025).

Furthermore, the South African public sector’s heavy reliance on short-term and fragmented digital skills programs highlights the overall failure to link digital inclusion with structural change. Agyemang and Bokpin (2025) argue that women’s digital empowerment should be integrated into models that consider social networks, risk tolerance, and time preference, elements often overlooked in technocratic program design. For example, while computer training centers may offer coding or data entry, they rarely address the challenges women face in dedicating uninterrupted time or managing household responsibilities (Munyeka and Maharaj, 2023). South Africa’s experience confirms that even well-funded initiatives may fall short if they do not tackle these fundamental inequalities.

Future research should focus on gender-specific interventions that address the unique challenges women encounter in the public sector. Studies should investigate the long-term impacts of digital literacy programs on women’s engagement in the digital economy and evaluate the effectiveness of stakeholder collaborations in promoting gender digital inclusion. Additionally, research into cultural barriers hindering women’s digital empowerment is essential, especially in contexts where patriarchal norms are deeply embedded. Moreover, future studies should analyze the role of public-private partnerships in advancing digital empowerment for women, emphasizing how these collaborations can be better coordinated to develop sustainable solutions.

## 5 Conclusion

The current study examines the barriers to female digital empowerment in the South African public sector. It investigates how government actions combined with stakeholder collaboration can promote greater inclusion in digital skills development programs for women. The findings from this systematic review show that, although policies are in place to promote gender equality in ICT, weak enforcement and inconsistent implementation hinder significant progress. Additionally, political instability and institutional obstacles—such as ingrained gender biases—further limit women’s participation in ICT roles. Socioeconomic barriers, especially in rural areas, highlight the need for investment in digital infrastructure and affordable access to technology.

The findings of this research suggest that despite these challenges, there is scope for improvement through targeted interventions and better coordination between the government and private stakeholders, including NGOs. These findings illuminate specific barriers women face in the public sector, which has been researched less than in the private sector.

One major limitation of this research is that it only examined peer-reviewed literature, which, although ensuring scholarly credibility, may restrict the scope of analysis. Valuable context-specific information from grey literature such as policy briefs, NGO reports, and grassroots studies was not included, despite being useful for documenting lived experiences and recent phenomena, particularly among marginalized and rural populations. Peer-reviewed journals are also susceptible to publication bias, favoring studies with positive, policy-aligned findings and thus underrepresenting critique or decolonial perspectives that are crucial for understanding structural differences in digital empowerment. Additionally, the time lag inherent in academic publishing could render some findings outdated in the rapidly evolving fields of ICT and digital governance, limiting the study’s ability to address new innovations or grassroots initiatives within the South African public sector. Nonetheless, despite these limitations, this study offers valuable insights into the structural and socioeconomic barriers limiting women’s full participation in the digital economy.

Key policy priorities shall focus on enhancing the implementation of existing legislation related to gender equality and making additional investments in digital skills to prepare for the long-term perspective. Further research is needed to assess the long-term impacts of these interventions and explore the role of public-private partnerships in promoting digital inclusion for women in other developing contexts.
